# The Effect of UV-C Pasteurization on Bacteriostatic Properties and Immunological Proteins of Donor Human Milk

**DOI:** 10.1371/journal.pone.0085867

**Published:** 2013-12-23

**Authors:** Lukas Christen, Ching Tat Lai, Ben Hartmann, Peter E. Hartmann, Donna T. Geddes

**Affiliations:** 1 School of Chemistry and Biochemistry, Faculty of Science, The University of Western Australia, Crawley, Western Australia, Australia; 2 Carag AG, Baar, Switzerland; 3 Perron Rotary Express Milk Bank, King Edward Memorial Hospital, Subiaco, Western Australia, Australia; 4 Centre for Neonatal Research and Education, The University of Western Australia, Crawley, Western Australia, Australia; University of Padova, Medical School, Italy

## Abstract

**Background:**

Human milk possesses bacteriostatic properties, largely due to the presence of immunological proteins. Heat treatments such as Holder pasteurization reduce the concentration of immunological proteins in human milk and consequently increase the bacterial growth rate. This study investigated the bacterial growth rate and the immunological protein concentration of ultraviolet (UV-C) irradiated, Holder pasteurized and untreated human milk.

**Methods:**

Samples (n=10) of untreated, Holder pasteurized and UV-C irradiated human milk were inoculated with *E. coli* and *S. aureus* and the growth rate over 2 hours incubation time at 37°C was observed. Additionally, the concentration of sIgA, lactoferrin and lysozyme of untreated and treated human milk was analyzed.

**Results:**

The bacterial growth rate of untreated and UV-C irradiated human milk was not significantly different. The bacterial growth rate of Holder pasteurized human milk was double compared to untreated human milk (p<0.001). The retention of sIgA, lactoferrin and lysozyme after UV-C irradiation was 89%, 87%, and 75% respectively, which were higher than Holder treated with 49%, 9%, and 41% respectively.

**Conclusion:**

UV-C irradiation of human milk preserves significantly higher levels of immunological proteins than Holder pasteurization, resulting in bacteriostatic properties similar to those of untreated human milk.

## Introduction

Human milk inhibits the growth of *Escherichia coli, Staphylococcus aureus* and *Candida* sp. [1,2]. The bacteriostatic properties of human milk are often attributed to the immunological proteins including lactoferrin, lysozyme and sIgA [3]. Lactoferrin is an iron-binding protein that reduces the availability of free iron required by iron-dependent pathogens such as *E. coli* and therefore inhibits their growth [4], as well as disrupting the bacterial cell membrane by binding to the lipid-A portion of lipopolysaccharides on the bacterial cell surface [5]. Lysozyme lyses the cell walls of most gram-positive bacteria such as *S. aureus* by catalyzing the hydrolysis of specific bonds between N-acetylglucosamine and N-acetylmuramic acid [6]. While lysozyme alone is bacteriostatic, an *in vitro* study showed that in presence of lactoferrin it is also bactericidal and can kill several gram-negative bacteria. The mechanism of action is not fully understood but it suggests that lactoferrin alters the gram-negative outer cell membrane, enabling lysozyme to break down the inner membrane of the bacteria [7]. Secretory IgA (sIgA) is an antibody, which is secreted specifically in response to the pathogens that the mother and infant are exposed to [8]. sIgA is highly resistant to the proteolytic activity of the digestive enzymes in the gastro-intestinal tract [9]. Therefore, its immunological function remains active and supplements the infant’s immune system [8]. Although sIgA has no known antimicrobial activity in isolation it enhances the antimicrobial activity of lactoferrin [10-12].

Donor human milk is the best alternative for preterm infants when mothers’ own milk is not available [13,14]. Donor human milk is pasteurized to prevent the potential risk for the transmission of pathogens from donor mothers to preterm infants. Holder pasteurization is a low-temperature long-time (LTLT) heat treatment widely used in milk banks. Bottles of human milk are heated in a water bath and held at 62.5°C for 30 minutes [15-17]. However, this heat treatment greatly reduces bioactive components of human milk, and immunological proteins are only partially preserved during this process. The retention rates after Holder pasteurization of sIgA, lactoferrin and lysozyme are ^~^72%, ^~^22%, and ^~^39%, respectively [18-20]. Reported retention rates are variable between studies and are likely due to different temperature profiles that the human milk was subjected to. Milk banking guidelines only define the holding temperature and holding time for Holder pasteurization. They do not state the full temperature profile, such as the heating and cooling period, used in the entire process of Holder pasteurization. The heating and cooling period in the Holder pasteurization process depends on many variables including milk volume, heat exchange surface to milk volume ratio, heat transfer rate of human milk depending on density and composition, heat transfer rate of the bottle depending on the material (glass or plastic) and wall thickness [21,22]. Therefore, these factors may partially explain the large variations in the retention of immune proteins of human milk reported.

Ultraviolet (UV) irradiation has been used for decades to reduce the microbial load in drinking water. It is currently an emerging disinfectant technology for other foods [23]. UV-C, specifically a wavelength between 250 and 270 nm, is highly germicidal [24,25]. At these wavelengths the pyrimidine and purine bases of the DNA absorb the UV-C energy causing chemical reactions. These reactions lead to photoproducts such as pyrimidine dimers, other pyrimidine adducts, pyrimidine hydrates, and may involve cross-links with proteins. On rare occasions breakages of DNA strands will occur [26]. However, UV-C irradiation also has the potential to photo-oxidize human milk proteins. Two types of photo-oxidation of proteins are known. Type 1 or direct oxidation were the protein structure (primarily side-chains) or the amino acids tryptophan, tyrosine, phenylalanine, histidine, cysteine and cysteine absorb the UV light resulting in the production of excited state species or radicals. Other amino acids are not absorbing UV light above 230 nm and are therefore not at immediate risk of damage at 254 nm [27]. Type 2 or indirect oxidation of proteins is caused by free radicals [27]. UV-C irradiation causes photolysis and subsequently generates reactive oxygen species (ROS) including hydrogen peroxide (H_2_O_2_) and free radicals such as superoxide anion (O_2_^-^), hydroxyl radical (^•^HO), peroxyl radical (ROO^•^) and ozone (O_3_) [25,28]. These generated ROS can then oxidize proteins and can impair their function.

Human milk is difficult to treat with UV-C due to its high absorption coefficient, which increases with the total solids concentration, thus limiting the penetration depth of the photons [26,29]. This limitation can be overcome with the application of a vortical flow of human milk around a UV-C source. With this method the content of vegetative bacteria in human milk can be reduced. Additionally, no loss of the enzymatic activity of alkaline phosphatase (ALP) and bile salt stimulated lipase (BSSL) was observed when treated with the UV-C dosage required for a 5-log_10_ bacterial reduction [29]. However, the effect of these UV-C dosages on the immunological proteins and bacteriostatic properties of human milk remain unknown. To consider UV-C irradiation as a viable alternative pasteurization method for donor human milk these effects are of high importance and need to be explored.

This study investigated the effect of UV-C irradiation on immunological proteins and the bacteriostatic properties of human milk, compared with the current Holder pasteurization method. Specifically, we characterized the growth rates of *E.coli* and *S. aureus* in Holder pasteurized and UV-C irradiated human milk and examined the effect of both treatment methods on the concentration of sIgA, lactoferrin and lysozyme. Additionally, we examined whether combinations of different dosages of UV-C and total solids concentration had an effect on the retention of sIgA, lactoferrin and lysozyme after UV-C irradiation.

## Methods and Materials

### Sample collection

Human milk was donated by ten lactating women (n=10 samples). The collection of the milk samples was approved by the Human Research Ethics Committee of the University of Western Australia (RA/4/1/2369). All donors gave written consent for their donations to be used in research and all samples were de-identified. Milk was donated frozen and stored in our laboratory in a -20°C freezer prior to the experiment.

### Sample Preparation

The human milk samples used in this study were the identical samples used in the study by Christen et al. [29]. Briefly, the total solids concentrations of human milk samples were adjusted to increase the concentration range and the macronutrient concentrations were; the protein ranged from 7 to 22 g/l; lactose 52 to 88 g/l and fat 10 to 81 g/l and the total solids ranged from 107 to 146 g/l within the 10 samples.

### Ultraviolet treatment

The ultraviolet pasteurization process and dosage levels used in this study were identical with the method used by Christen et al. [29]. Their experimental setup showed a 5-log_10_ bacterial reduction at 4863 J/l with a better retention of alkaline phosphatase and bile salt stimulated lipase than Holder pasteurization. Part of each human milk sample (380 ml; n=10) was transferred into a 400 ml PYREX glass beaker No. 1003. The human milk was exposed to UV-C (1.1 W) and samples were taken at different time points representing the different experimental exposure dosages (0, 2084, 3474 and 4863 J/l), portioned in to tubes and kept frozen at -80°C until the laboratory analyzes were performed.

### Holder pasteurization

Part of each human milk sample (1.6 ml; n=10) was transferred into a 2 ml polypropylene micro tube. Samples were then placed into a preheated water bath (CC-1, Huber GmbH, Offenburg, Germany) and brought to 62.5°C and held at this temperature for 30 minutes, then immediately cooled down in an ice bath until the temperature reached 4°C. The samples were kept frozen at -80°C until the laboratory analyzes were performed.

### Bacteriostatic properties analysis

*Escherichia coli* K12 (ATCC1498, Southern Biological, Nunawading, Vic, Australia) and *Staphylococcus aureus* (ATCC6538, American Type Culture Collection Inc, Manassas, VA, USA) were cultured in nutrient broth (Nutrient Broth No. 2, Oxoid Australia Pty Ltd, Adelaide, SA, Australia) overnight at 37°C. After enumeration using the optical density method with previously constructed standard curves [30], the cultures were diluted and inoculated into untreated, UV-C irradiated and Holder pasteurized samples to bacterial loads of *E. coli* and *S. aureus* of 4000 CFU/ml (colony forming units per millilitre). All samples were plated in duplicate onto eosin methylene blue agar (selective for *E. coli*) and mannitol salt agar (selective for *S. aureus*) (Oxoid Australia Pty Ltd, Adelaide, SA, Australia) immediately after inoculation and after 2 hours of incubation at 37°C [31,32]. The plates were then incubated at 37°C overnight and the numbers of colonies enumerated to determine the bacterial number based on colony forming units per millilitre (CFU/ml).

### Measurement of protein retention

The sIgA and lactoferrin content of the human milk samples were quantified by the sandwich Enzyme-linked immunosorbent assay (ELISA) method as previously described [18]. All antibodies and standards were purchased from MP Biomedicals Australasia (New South Wales, Australia). The recovery assay for sIgA was 85±4% (n=6) and for lactoferrin 82±9% (n=6). Lysozyme content of human milk samples was quantified using a Human Lysozyme enzyme immune assay (EIA) kit (Biomedical Technologies, Inc., MA) according to manufacturer’s instructions.

### Statistical analysis

Data analysis was performed using R 2.15.1 for Mac OS X (The R Core Team) [33]. Packages nlme [34], multcomp [35], and lattice [36] were used for linear mixed models, multiple comparisons of means, and graphical representation of the data, respectively. *P*<0.05 was considered to indicate significant difference in all analyzes. Results are presented as mean ± standard deviation (SD) values unless otherwise stated. 

Linear mixed effects models were used to determine i) If bacterial growth rate was different within the 2 species; ii) If the bacterial growth rate was different for different treatment methods; iii) If the retention of a) sIgA, b) lactoferrin and c) lysozyme was different for different treatment methods. Individual human milk sample was considered as the random effect. Tukey's HSD was used for post-hoc testing to identify which treatments were significantly different.

Linear regression was used to determine if the retention of i) sIgA, ii) lactoferrin and iii) lysozyme was related to total solids concentration and to determine if bacterial growth rate was related to the concentrations of i) sIgA, ii) lactoferrin, iii) lysozyme and iv) sum of three proteins after accounting for treatment (untreated, UV-C irradiated and Holder pasteurized). Although the data for this analysis comes from related samples, analysis of variance comparison of regression and linear mixed effects models with the same fixed effects found no individual sample effects.

Two-way and three-way interaction in ANOVA was used to determine if sIgA, lactoferrin or lysozyme is different across the different treatments of the bacterial multiplication rate.

## Results

In order to evaluate the impact of different milk treatments on bacterial growth, we assessed the growth rate of *E. coli* and *S. aureus* during 2 hours of incubation by sampling at inoculation and 2 hours post incubation at 37°C in untreated human milk and pasteurized with UV-C and Holder. There was no difference in the growth rate of the two species over the two-hour incubation time in all of the samples (p=0.69), therefore, they were grouped together for the remaining statistical analyses.

### Untreated Human Milk

Bacteria incubated in untreated human milk had a growth rate of 2.7±1.1 fold per hour. The concentrations of sIgA, lactoferrin and lysozyme in untreated human milk were 3.45±0.70, 3.00±0.83, and 0.024±0.015 g/l, respectively (Table 1).

**Table 1:** Concentration of immunological proteins of untreated, UV-C irradiated and Holder pasteurized samples.

	**sIgA**		**Lactoferrin**		**Lysozyme**	
**Treatment**	**concentration [g/l]**	**retention [%]**	**concentration [g/l]**	**retention [%]**	**concentration [g/l]**	**retention [%]**
**untreated**	3.45±0.70	100%	3.00±0.83	100%	0.024±0.015	100%
**UV-C 2084 J/l**	3.29±0.65	95±5%	2.86±0.87	95±6%	0.021±0.013	91±7%
**UV-C 3474 J/l**	3.22±0.64*	94±4%	2.81±0.87	93±10%	0.019±0.011*	84±10%
**UV-C 4683 J/l**	3.07±0.63**	89±4%	2.63±0.86	87±11%	0.017±0.009**	75±9%
**Holder**	1.67±0.32**	49±3%	0.26±0.14**	9±4%	0.010±0.008**	41±14%

*p<0.05, **p<0.001 denotes significance with respect to untreated samples

(mean±SD, n=10).

### UV-C irradiated human milk

**Figure 1:**
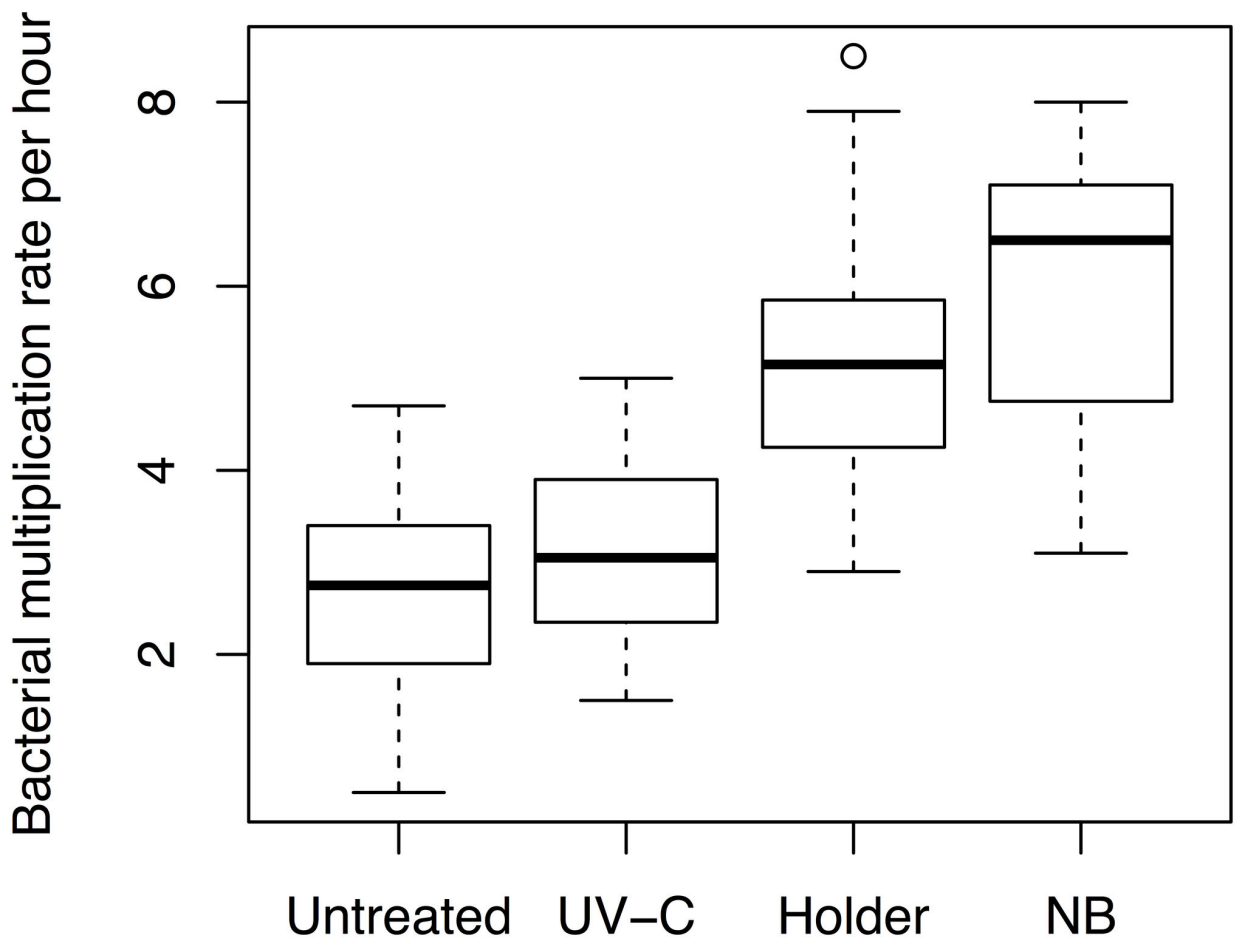
Bacterial growth for untreated, UV-C irradiated and Holder pasteurized human milk and nutrient broth (NB).

**Figure 2:**
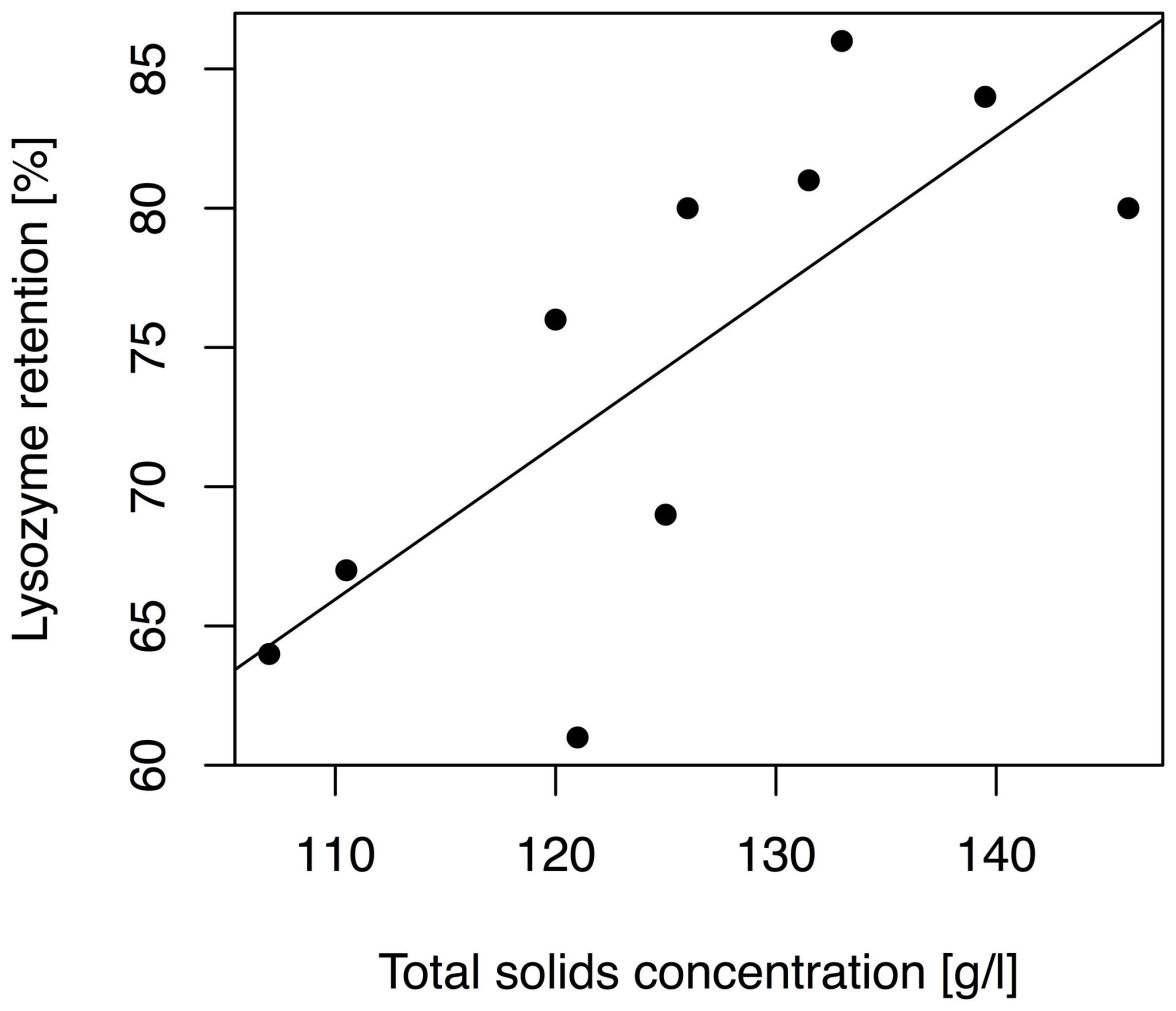
Relationship between total solids concentration and lysozyme retention in UV-C irradiated human milk. Full legend: Relationship between the total solids concentration and the retention (%) of lysozyme in UV-C irradiated human milk at a dosage of 4683 J/l.

Bacteria incubated in UV-C irradiated human milk (4683 J/l) had a growth rate of 3.1±0.9 fold per hour, which was not significantly different to untreated milk (p=0.75) (Figure 1). The samples irradiated with a dosage of 3474 J/l of UV-C had a significantly lower concentration of sIgA (p=0.036) and lysozyme (p=0.032) compared to the untreated sample, when treated with 4683 J/l the significance increased to p<0.001 for both proteins. Dosages used did not significantly reduce the concentration of lactoferrin (p=0.28) (Table 1). Greater lysozyme retention was seen with higher concentrations of total solids (p<0.001) (Figure 2). For each additional g/l of total solids in human milk the retention of lysozyme was increased by 0.55 percentage points. No significant correlation was found between the total solids concentration and sIgA (p=0.45) or lactoferrin (p=0.23) retention after UV-C irradiation.

### Holder pasteurized human milk

Bacteria incubated in Holder pasteurized human milk had a growth rate of 5.1±1.4 fold per hour. The bacterial growth rate of Holder pasteurized human milk was significantly higher than both untreated and UV-C irradiated human milk (p<0.001) (Figure 1). Significant reductions in sIgA, lactoferrin and lysozyme were observed after Holder pasteurization (p<0.001) (Table 1). No correlation was found between the concentration of total solids and the retention of immunological proteins during Holder pasteurization (p=0.76).

### Nutrient broth

Bacteria incubated in nutrient broth had a growth rate of 5.9±1.5 fold per hour, which was not significantly different from that of Holder pasteurized human milk (p=0.13), but was significantly higher than both untreated and UV-C irradiated human milk (p<0.001) (Figure 1).

### Bacterial growth rate in relation to immunological proteins

**Figure 3:**
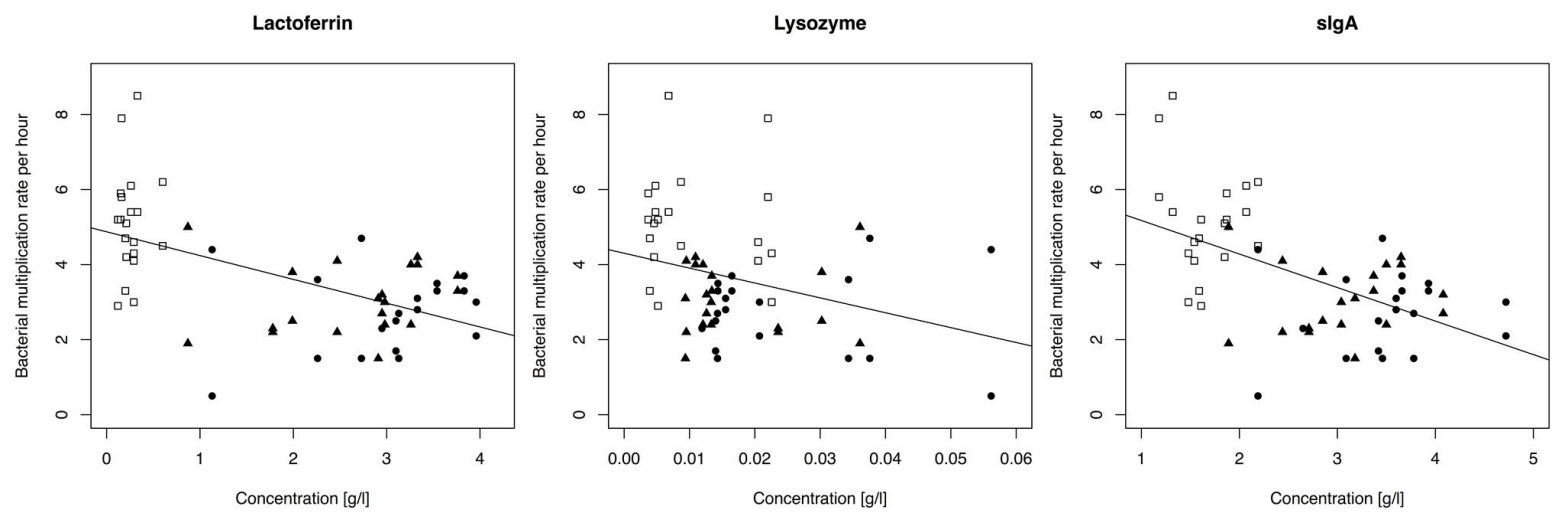
Relationship between bacterial multiplication rate per hour and concentration of lactoferrin, lysozyme and sIgA. Full legend: Relationship between bacterial multiplication rate per hour and concentration of lactoferrin, lysozyme and sIgA in human milk samples (●untreated, ▴UV-C irradiated, □Holder pasteurized human milk).

Taking all treatments (untreated, UV-C irradiated, Holder pasteurized) into account, bacterial growth rate per hour was lower with higher concentrations of lactoferrin (p<0.001), lysozyme (p=0.019) and sIgA (p<0.001) (Figure 3). Taking each treatment separately, no correlation was found between bacterial growth rate and lactoferrin (p>0.50), lysozyme (p>0.91) and sIgA (p>0.60). Untreated and UV-C irradiated samples showed no three-way and two-way interactions between bacterial growth rate and sIgA:lactoferrin (p=0.41), lactoferrin:lysozyme (p=0.60), sIgA:lysozyme (p=0.71) and sIgA:lactoferrin:lysozyme (p=0.19). The same analyzes with the Holder pasteurized samples found a significant two-way interaction between bacterial growth rate and lactoferrin:lysozyme (p=0.016) but no other interactions, sIgA:lactoferrin (p=0.83), sIgA:lysozyme (p=0.22) and sIgA:lactoferrin:lysozyme (p=0.30).

Human milk samples showed a high correlation between the concentration of sIgA and lactoferrin (p<0.001) but no significant correlation was found between sIgA and lysozyme (p=0.35) and lactoferrin and lysozyme (p=0.25).

## Discussion

This study found that UV-C pasteurized human milk had higher retention rates of immunological proteins than Holder pasteurized human milk. Furthermore, the bacterial growth rate of UV-C pasteurized human milk and untreated human milk were similarly, unlike Holder pasteurized human milk where the bacteria multiplied significantly faster.

Human milk irradiated with a UV-C dosage of 4683 J/l had a higher retention rate of lactoferrin, lysozyme and sIgA (87%, 75% and 89%, respectively) compared with Holder pasteurization with 9%, 41% and 49%, respectively (Table 1). The finding that UV-C irradiation caused less damage to proteins than thermal pasteurization agrees with a previous study conducted with whey protein solutions [37]. We have recently shown that a dosage of 4863 J/l reduces vegetative bacteria by 5-log_10_ in human milk with a high total solids content without the loss of BSSL, ALP and fatty acids [29]. However, we also found a positive linear relationship between the dosage required for bacterial reduction and the total solid content in human milk [29]. Therefore we investigated in this study the interaction of total solids and dosage on lactoferrin, lysozyme and sIgA. The retention of lactoferrin, lysozyme and sIgA was higher when treated with lower UV-C dosages (Table 1). Additionally, we also found a positive linear relationship between lysozyme and total solid content. It appears that a high total solids concentration provides a protective effect against loss of lysozyme (p<0.001) (Figure 2). It is possible that increased total solids content provides optical protection of UV-C photons whereas a low total solid content decreases the absorption coefficient of human milk. Therefore, photons can travel farther and more lysozyme is exposed. We speculate that UV-C irradiation caused a type 1 photo-oxidation (direct absorption of photons by the protein structure or amino acids) of lysozyme. This speculation is supported by the fact that lysozyme contains 5 of the 6 amino acids (tryptophan, tyrosine, phenylalanine, histidine and cystine) [38] that are absorbing at this wavelength [27]. In contrast we have not found a correlation between the concentration of total solids and the retention of sIgA (p=0.45) and lactoferrin (p=0.23). We can therefore speculate that the damage was not caused by direct photon absorption but rather a type 2 photo-oxidation (indirectly oxidized by ROS). Further, since the dosage required to reduce vegetative bacteria is lower for milk with low total solids concentration [29] it would be possible to apply a lower dosage and therefore, increase the retention of immunological proteins whilst still reducing bacteria to acceptable levels. 

The growth rate of *E. coli* and *S. aureus* in human milk irradiated with a UV-C dosage of up to 4683 J/l was not significantly different than in untreated human milk (Figure 1). In contrast the bacterial growth rate per hour of *E. coli* and *S. aureus* in Holder pasteurized human milk was double that of untreated human milk and was not significantly different to nutrient broth (Figure 1), which is optimized for rapid growth of these two species of bacteria. These results are similar to the findings of Silvestre et al. [31] and Van Gysel et al. [32] where a decrease in antimicrobial activity was reported after Holder pasteurization. In contrast to our study, these authors found that the Holder pasteurized human milk still had a better antimicrobial activity than both of their controls peptone broth and tryptic soy broth. The difference between findings may be due to the use of different bacterial strains and the different bacterial growth rates in nutrient, peptone and tryptic soy broth. Additionally, our Holder pasteurized human milk had a lower retention of lactoferrin of 9% compared to previous reports of between 20% and 43% [19,20]. The retention of lysozyme (41%) and sIgA (49%) in our study were consistent with lower values reported in the previous literature [19,20]. Silvestre et al. [31] and Van Gysel et al. [32] may have had higher retention of immunological proteins due to different pasteurization conditions caused by a different temperature profiles the human milk was exposed to. The heating and cooling time may have been different due to different treatment volumes, different bottles with different diameter to height ratio, different materials and wall thicknesses. Nevertheless, the findings of all three studies indicate that holder pasteurized donor human milk has a significantly faster bacterial growth rate than untreated human milk due to the reduced antimicrobial activity, and therefore, must be handled with strict hygienic practice to ensure minimal risk of bacterial growth during dispensing and delivery in the neonatal intensive care unit (NICU). Cohen et al. [39] demonstrated that without the addition of additives, pasteurized donor human milk handled in 'standard clinical fashion' in a NICU, remains bacterial culture negative for 24 hours after thawing. However, it is almost universal practice to supplement human milk for preterm infants with human milk fortifier (HMF), which may not be sterile. HMF could therefore be a potential source of bacteria that might multiply more quickly in Holder pasteurized donor human milk than untreated or UV-C irradiated human milk. Further study in this respect is clearly warranted.

Lactoferrin and lysozyme are considered to exert the majority of antimicrobial activity due to their high abundance in human milk [6,40,41]. Lactoferrin and lysozyme constitute approximately 22% and 0.6% of the total protein content in human milk respectively [6]. Furthermore, the concentration of lysozyme is around 3000 times higher than in bovine milk [41]. Our results show that the multiplication rates of *E. coli* and *S. aureus* have a strong inverse correlation to the concentration of lactoferrin (p<0.001), lysozyme (p=0.019) and sIgA (p<0.001) (Figure 3). Although sIgA alone is not known to have antimicrobial activities in human milk we found an increased inhibition of bacterial growth with higher concentrations of sIgA overall treatments. However, we also found that high levels of sIgA were associated with high levels of lactoferrin (p<0.001) therefore lactoferrin may be responsible for the reduced bacterial growth rather than sIgA. 

Considering the effect of treatment method (untreated, UV-C irradiated and Holder pasteurized) if untreated and UV-C irradiated samples are taken into account no correlation or interactions were found between the bacterial growth rate and lactoferrin, lysozyme and sIgA. In contrast the Holder pasteurized samples alone showed a correlation between the bacterial growth rate and an interaction effect of lactoferrin and lysozyme (p=0.016). This finding correlates with a previous study that found enhanced antimicrobial activities against gram-negative bacteria when both, lactoferrin and lysozyme are present [7]. However, this effect was not evident in untreated and UV-C irradiated human milk. It may be possible that the higher retention rates of lactoferrin, lysozyme and sIgA with untreated and UV-C irradiated human milk (Table 1) have inhibited bacterial growth making it difficult to elucidate the relationship shown in the Holder treated samples. Our samples (untreated and UV-C irradiated) showed an inverse correlation of lactoferrin and lysozyme concentration, however, in Holder pasteurized samples lactoferrin was reduced to a much greater extent than lysozyme. This different reduction could have unmasked the synergistic effect of lactoferrin and lysozyme. Furthermore, the negative finding in the untreated and UV-C irradiated human milk suggests a possible threshold effect above which antimicrobial activity does not change with increased immunological protein concentration. Alternatively, the association may have been masked by the lack of samples with lower concentrations of immunological proteins in the untreated and UV-C irradiated group, which could be achieved by exposing human milk to higher UV-C dosages and increasing numbers of samples in the untreated group to extend variability. Similarly, concentrations of immunological proteins in the Holder pasteurization group could also be improved by exposing the milk to a different temperature profile as suggested by Czank et al. [18] or Wills et al. [42]. These experiments will further explore the relationship between methods of pasteurization and the bacteriostatic properties of human milk. Other human milk components with antimicrobial properties include lactoperoxidase [43], polypeptides released from α-lactalbumin [44] and possibly N-acetyl-ß-D-glucosaminidase [45] may also play a role in reducing bacterial growth. Recently, an additional 700 proteins have been identified in human milk [46,47]. These proteins are low in concentration and approximately a quarter of them are considered to have an immune response function [47] although their potency and activity in respect of inhibition of bacterial multiplication are unknown. With such large numbers of immune factors in human milk, it is difficult to determine the actual proportion of antimicrobial activity afforded by lactoferrin, lysozyme and sIgA without considering the antimicrobial activity from other immunological components including synergistic effects of numerous components. Nevertheless, in terms of all antimicrobial components combined we can speculate that the dosages of UV-C irradiation used in this study do not exert a major influence on their activity since the bacterial growth rate of UV-C irradiated human milk was not significantly different to untreated human milk.

Increasing the retention of immunological proteins of pasteurized donor human milk is important due to their role in reducing health complications of preterm infants in the NICU [3,48-54]. Studies have shown a decreased incidence of inflammation and local and systemic infections such as urinary tract infections, invasive fungal infections (IFI), late onset sepsis (LOS) and necrotizing enterocolitis (NEC) although some studies particularly those of NEC are inconclusive due to the multifactorial nature of the disease. One study, however, has shown the incidence of late onset sepsis (LOS) in very low birth weight (VLBW) infants was greater for infants fed with artificial formula (26%; n=88) and holder pasteurized donor human milk (23%; n=78) compared to mothers’ own milk (16%; n=70) [55]. The incidence of LOS in the donor human milk group is likely due to the inclusion of 21% of the infants that also received preterm formula due to poor weight gain and this aberration was not accounted for statistically. Nevertheless another study of VLBW infants (n=321) has shown that the supplementation of 100 mg/d of bovine lactoferrin to mothers’ own milk significantly reduced the incidence of LOS from 17.3% to 5.9% [53]. These results indicate that the incidence of LOS might in part be related to the concentration of lactoferrin in the human milk. Therefore, future studies would need to take into account the complexity of human milk composition with regard to immunological proteins and the method by which the human milk is treated. 

## Conclusion

This study demonstrates that UV-C irradiation results in better overall preservation of the bacteriostatic properties of human milk than Holder pasteurization. Specifically the individual immunological proteins sIgA, lysozyme and lactoferrin had significantly higher retention rates after UV-C irradiation compared to Holder pasteurization. However due to the complex nature of human milk it is difficult to accurately determine the individual contribution of these major immunological proteins on the bacteriostatic properties, and some effects may be synergistic. This finding has implications for the development of novel processing technologies for donor human milk banks with the aim of increasing product quality. The finding of increased bacterial growth rate in holder pasteurized human milk may also have implications for current food handling practices in NICU that fortify holder pasteurized donor human milk with non sterile commercial human milk fortifiers.
